# Supplementary figures and images for: Anti-inflammatory activity of diindolylmethane alleviates Riemerella anatipestifer infection in ducks
Source: PLoS One. 2020 Nov 11;15(11):e0242198. doi: 10.1371/journal.pone.0242198 (PMC7657562; doi:10.1371/journal.pone.0242198)

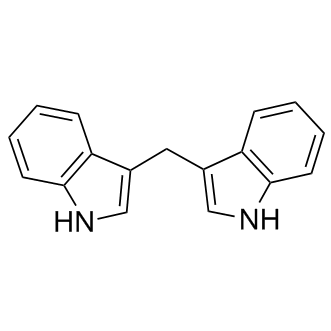


**Supplementary Figure 1**. Chemical structure of 3,3’-diindolylmethane (DIM).

Supplement: S1 Fig — (DOCX) [file pone.0242198.s001.docx]
