# Supplementary material for: Anti-inflammatory activity of diindolylmethane alleviates Riemerella anatipestifer infection in ducks
Source: PLoS One. 2020 Nov 11;15(11):e0242198. doi: 10.1371/journal.pone.0242198 (PMC7657562; doi:10.1371/journal.pone.0242198)
Supplement: Attachment — Submitted filename: Anti-inflammatory activity of diindolymethane alleviates Riemerella anatipestifer infection in ducks.docx [file pone.0242198.s002.docx]

This study found DIM alleviates RA infection in ducks, by down-regulated expression of inflammatory cytokines such as INF-γ, IL-10, IL-17A, IL-6, IL-1β and IL-17F.

Q1. DIM has effects on the expression of these cytokines, also DIM was associated with signaling pathways such as JNK, p38, NF-κB, AP-1, and FAK. Which signal pathway is mainly? Also can you explain that how DIM kills bacteria on ducks?

Q2. On the Fig.2A, in the RA infected group from liver, it displays lower bacterial load on three ducks, even close to zero. Did it mean that ducks clear bacterium by themselves, but has nothing to do with DIM treatment? Maybe you increase the number of ducks.

Q3. On the Fig.2B, only 14% survival rate is contributed by DIM, is there a signification on the clinical treatment? Maybe we increase infectious dose, whether the survival rate of ducks infected with RA and DIM treatment is lower same as survival rate of ducks infected with RA?

Q4. All cytokines detected are by RT-qPCR method, can you detect them by other methods, such as ELISA, WB?

Suggestion 1. Can you move Figure 1A to the supplementary results.

Suggestion 2. The Fig.6D was wrong, can you correct it.
